# Exploration of the effects of Tai Chi practice on lower limb corticomuscular coherence during balance-demanding virtual reality conditions in older adults

**DOI:** 10.3389/fnagi.2025.1554000

**Published:** 2025-06-23

**Authors:** Yang Hu, Elizabeth T. Hsiao-Wecksler, Manuel E. Hernandez

**Affiliations:** ^1^Department of Kinesiology, San Jose State University, San Jose, CA, United States; ^2^Department of Mechanical Science and Engineering, Grainger College of Engineering, University of Illinois Urbana Champaign, Urbana, IL, United States; ^3^Department of Biomedical and Translational Sciences, Carle Illinois College of Medicine, University of Illinois Urbana Champaign, Urbana, IL, United States

**Keywords:** EEG, EMG, virtual reality, Tai Chi, corticomuscular coherence

## Abstract

Tai Chi practice has been widely adopted to improve balance and prevent falls in older adults. However, the neural mechanisms underlying the benefits of Tai Chi are difficult to evaluate during traditional balance assessments. The goal of this study was to evaluate the effects of Tai Chi and healthy aging on corticomuscular coherence (CMC) while standing in virtual balance-demanding environments. We recorded neural, muscular, and behavioral data in an immersive virtual reality environment while implementing sensory and mechanical perturbations to introduce high postural threats. Through the acquisition of electroencephalography and electromyography signals, we examined β and γ CMC changes in frontal, central, parietal, and occipital cortical areas and ankle plantar- and dorsi-flexors in older adults (*n* = 10), older adults with Tai Chi practice (*n* = 10), and young adults (*n* = 10). The results showed that older adults have higher γ CMC in comparison with Tai Chi practitioners and young adults as evaluated by the magnitude square coherence. Increased β and γ CMC correlated with decreased mediolateral postural sway in older adults, while young adults demonstrated the opposite relationship. Furthermore, lower tibialis anterior and soleus β CMC were found in older adults during ground conditions compared to Tai Chi practitioners and young adults. The results demonstrate the effects of aging and Tai Chi on CMC during balance-demanding standing tasks, and the potential application of the novel system to quantify cortical and muscular adaptation after rehabilitation.

## 1 Introduction

With a rapidly increasing number of individuals over 65 years of age globally, more people face functional impairment associated with the aging process, such as reductions in balance and increasing falls (Sturnieks et al., 2008). Balance control is defined as the act of maintaining, achieving or restoring a state of balance that may involve either a fixed support or a change in support response (Pollock et al., 2000). Balance control requires a complex interplay with and between the cortical, sensory, and motor systems (Dieën and Pijnappels, 2017). At the same time, Tai Chi practice has been widely used as a non-pharmacological approach to improve postural control and prevent falls in older adults (Li et al., 2019). However, the neural mechanisms underlying Tai Chi practice benefits remain to be explored (Zhu et al., 2010).

Tai Chi practice has been well documented in changing neuromuscular functions in older adults, as measured by electromyography (EMG), and provides a potential mechanism for the improvement of postural control abilities (Hu et al., 2021). These neuromuscular changes include decreased reaction time, earlier muscle onset time, H-reflex modulation, increased muscle strength, and improved muscle coordination pattern (Hu et al., 2021). Meanwhile, previous studies also suggest alterations in cortical structure and functional neural activity after Tai Chi practice, which give rise to beneficial neurological changes in the human brain (Pan et al., 2018). Significant changes were reported on subjects' cortical thickness, functional connectivity, homogeneity of the brain, and executive network functions after Tai Chi intervention (Pan et al., 2018). Specifically, in a study that compared Tai Chi experts and novices, they found significantly higher β-wave amplitude in Tai Chi experts than Tai Chi novices (Liu et al., 2003). In task-switch tests under homogenous and heterogeneous conditions, long-term Tai Chi practitioners demonstrated significantly larger P3 event-related potential than sedentary older adults, which were more like young adults' P3 (Fong et al., 2014). Tai Chi practice may influence cortical control processes under energetic constraints, as seen in older adults (Schumann et al., 2022; Tomporowski, 2008) and allow for task-specific increases in cortical resources to meet increased balance demands. Furthermore, while hand beta corticomuscular coherence (CMC) has been found to be altered with increased Tai Chi practice (Kerr et al., 2016), examining CMC while standing in complex virtual reality environments might provide additional insight into the mechanism of the Tai Chi practice's benefits on improving postural control performance.

Virtual reality allows for precise and synchronized multimodal stimuli, such as visual and auditory cuing of elevated heights, that has been demonstrated to elicit physical and physiological responses comparable to real-world exposure (Cleworth et al., 2012). Virtual height exposure can be combined with mechanical perturbations, to create immersive balance-demanding tasks that may be more generalizable to real-world conditions and detection of psychological contributions (Bzdúšková et al., 2022) , than traditional clinical evaluations of postural control, such as a Romberg test (Black et al., 1982) or computerized dynamic posturography (Baloh et al., 1994).

Coherence between electroencephalography (EEG) and EMG, defined as cross spectra normalized by auto spectra (Mima and Hallett, 1999), calculated using is thought to reflect corticospinal coupling between cortical areas and muscle motor units (Mima and Hallett, 1999; Negro and Farina, 2011). The coherence between EEG and EMG, which are consistent with the conduction time between the motor cortex and the respective muscle (Gwin and Ferris, 2012), can be evaluated by CMC phase lags. Meanwhile, the frequency band where CMC is most prominent is affected by the type of motor task. Specifically, β range (13–30 Hz) CMC between cortical area and lower limb muscles has been documented during static force output and isometric contractions, and γ range (31–45 Hz) CMC has been documented during isokinetic contractions (Gwin and Ferris, 2012).

Aging is associated with neuromuscular changes that impair corticomuscular communication (Yoshida et al., 2017), including decreased white matter volume (Salat et al., 2005) and motor neuron recruitment (Tomlinson and Irving, 1977). Older adults have demonstrated lower CMC in voluntary movements or isometric contraction of the upper extremity, compared to young adults, with lower CMC associated with lower strength (Bayram et al., 2015; Kamp et al., 2013). Furthermore, decreases in β (20–30) and lower γ (30–40) CMC have been observed in adults with amyotrophic lateral sclerosis and stroke, relative to healthy controls (Proudfoot et al., 2018; Fang et al., 2009). However, increased β band CMC in older adults, relative to young adults, has been found in dual task conditions, where concurrent cognitive and fine motor tasks are performed (Johnson and Shinohara, 2012). Furthermore, increased β CMC has been associated with increased accuracy in dual task conditions but only in young adults (Johnson and Shinohara, 2012). While less is known about gamma frequency changes with aging, increased γ-range CMC has been associated with increased demands in sensorimotor integration processes (Omlor et al., 2007) that may be expected in older vs. younger adults.

Considering the inconsistencies observed in the examination of the effects of aging on CMC and the few studies that have examined the neuromuscular basis of Tai Chi practice benefits on postural control, the purpose of this study was to evaluate the effects of Tai Chi practice and healthy aging on cortical and neuromuscular function, as evaluated by CMC, while standing in realistic and challenging environments. This study investigated the connection between cortical activation, lower limb muscle activities, and underlying postural control in novel standing balance conditions to achieve this goal. We hypothesized that (1) compared to young adults and older adults with Tai Chi practice, older adults would demonstrate significantly lower β CMC and higher γ CMC, specifically in less challenging conditions, given decreases in β CMC seen in older adults (Bayram et al., 2015; Kamp et al., 2013) and increases in γ CMC seen when tasks require increased sensorimotor integration (Omlor et al., 2007), (2) β CMC would be significantly correlated with the center of pressure indices in conditions that required isometric contractions, and that (3) γ CMC would be significantly correlated with the center of pressure indices in conditions that required non-isometric contractions , given observed changes in β and γ range CMC in lower extremity isometric and isotonic exercises (Gwin and Ferris, 2012).

## 2 Methods

### 2.1 Study participants

Ten healthy young adults, ten healthy older adults, and ten healthy older adults with Tai Chi practice experience were recruited for this study (Table 1). An a-priori power analysis using G-power, based on the effect of Tai chi on cortical electrical activity across central and occipital channels during a postural control task in beta oscillatory activity (Liu et al., 2003), which demonstrated a medium effect size, *f* = 0.25, was used to establish the 30 person sample size for a within-between group interaction for F tests at 80% power at *alpha* = 0.05 for three groups and four repeated measures. Participant inclusion criteria were as follows: (1) right-handed; (2) over 18 years old; (3) free of chronic or acute neurological conditions, such as Parkinson's disease, Huntington's disease, stroke, epilepsy, and seizures; (4) and free of severe heart conditions, such as history of heart attack, heart failure, and angina; (5) no lower limb injury in the past 3 months; and (6) normal or corrected vision. Exclusion criteria were as follows: (1) cognitive dysfunction, determined by a Modified Telephone Interview for Cognitive Status (TICS-M) questionnaire score lower than 18 (Cook et al., 2009); (2) severe chronic pain that limits physical activity; or (3) severe motion sickness. Furthermore, younger adults were limited to 18–30 years, while older adults were limited to individuals over 65 years of age, and older adults with Tai Chi practice were over 65 years of age while currently practicing Tai Chi and having Tai Chi practice of at least 2 h per week in the past 16 weeks. Once included in the study, all participants signed a written informed consent form. The protocol and procedures have been reviewed and approved by the Institutional Review Board of the University of Illinois at Urbana Champaign (IRB Protocol No. 15317, Approved 08/15/2023).

**Table 1:** Characteristics of study participants.

**Characteristic**	**Young adult**	**Older adult**	**TCP**
	**Mean (SD)**	**Mean (SD)**	**Mean (SD)**
Age	20(2)	71(5)*	76(6)^*^
Sex (F/M)	5/5	6/4	6/4
BMI	24.30 (6.03)	24.86 (5.55)	22.81 (2.53)
TCP hours	0	0	61–9,360
TMT-A	20.86 (6.27)	27.71 (7.78)	34.00 (6.99)^*^
TMT-B	45.80 (19.19)	57.05 (16.93)	65.30 (22.36)
FES-I	17.40 (2.12)	19.20 (3.01)	21.30 (4.08)^*^
Acrophobia	29.55 (34.58)	20.40 (13.28)	35.30 (21.40)
miniBEST	26.00 (1.25)	24.90 (1.97)	23.50 (2.76)^*^

SD, standard deviation; BMI, body mass index; TCP, Tai Chi practice; TMT, trail making test; FES-I, falls efficacy scale-international.

^*^Cohort difference relative to young adults, *p* < 0.05.

### 2.2 Experimental paradigm

**Figure 1:**
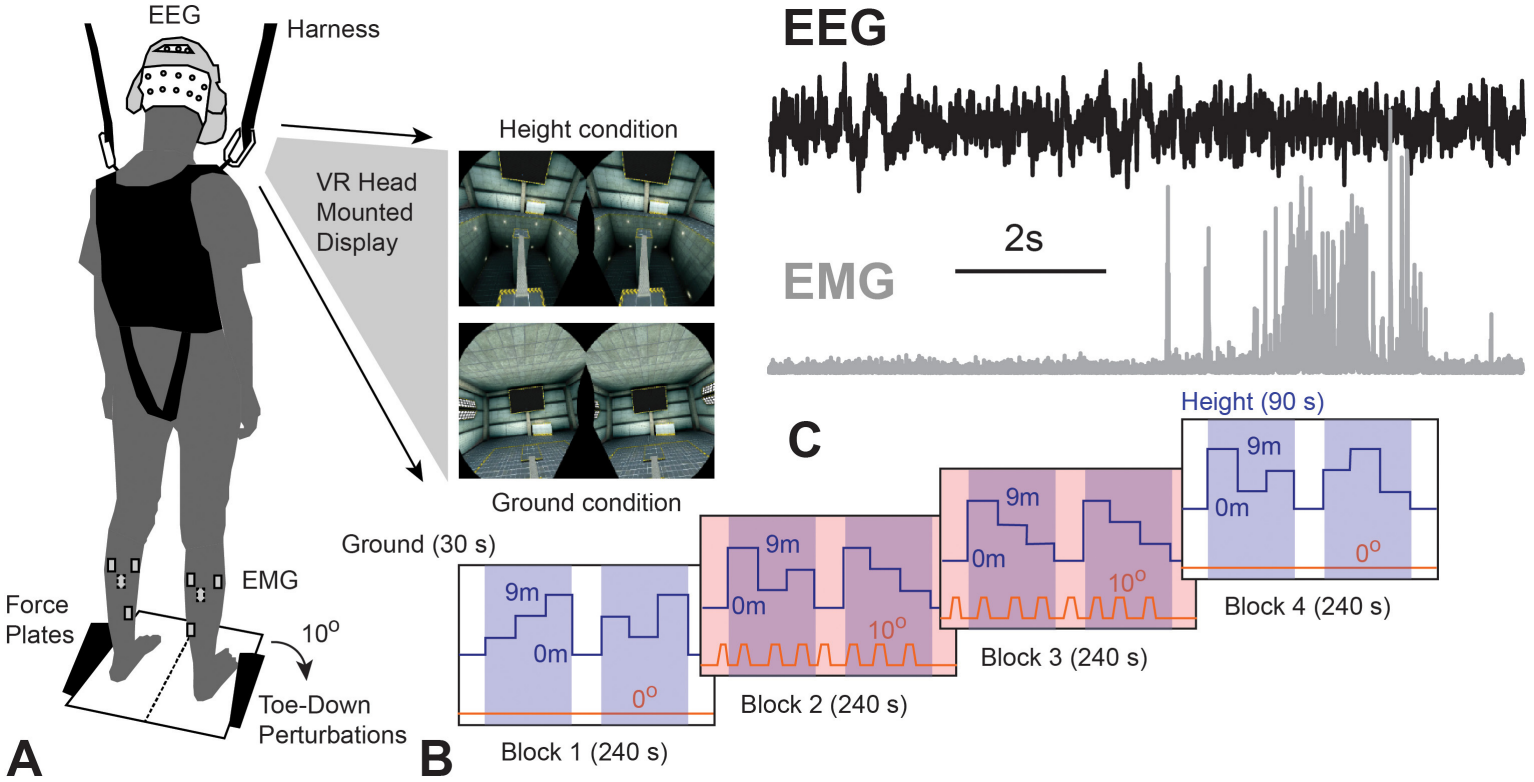
**(A)** Experimental setup and example of VR environment; **(B)** schematic of experimental protocol, with white regions (30 s in duration) representing ground conditions, blue shaded regions (90 s in duration) representing height conditions, and orange shaded regions (240 s in duration) representing perturbations in blocks 2–4; **(C)** sample electroencephalography (EEG) and electromyography (EMG) segment during perturbation in a representative older adult with Tai Chi practice.

This study consisted of a single session cross-sectional experimental design. Ground conditions, 30 s in duration, were provided before 90 s height conditions within each 240 s block. Four counter-balanced blocks consisting of no perturbation (blocks 1 and 4) or perturbation (blocks 2–3) conditions were provided (Figure 1). To incorporate sensory and mechanical perturbations, VR was used to provide a simulation of virtual height changes, which introduced sensory perturbations and integrated an actuator to provide mechanical perturbations. Participants were asked to stand as still as possible without taking steps while EEG, EMG, and center of pressure (COP) data were recorded. Multimodal data were synchronized using Vizard (WorldViz, Inc) scripts that provided TTL pulses for EEG data, initiated force plate data collection at the start of each block, and use of a specific pseudorandomized perturbation profile, as previously described (Widdowson et al., 2016). Additional functional tests and questionnaires were conducted to control for potentially confounding factors.

### 2.3 Virtual reality height control test

The virtual reality height control test (VR-HCT) combined sensory input and mechanical perturbation through the use of a head-mounted display (HTC Vive, HTC Corp.). The mechanical perturbations were induced by the SMART EquiTest-Clinical Research System (SECRS, Neurocom, a division of Natus) as a 10-degree, 2.5 s, toe-down perturbations. This perturbation was similar to the perturbations incorporated in the clinical adaptation test (ADT), designed to engage the brainstem and cortex. An industrial lift in a barren factory floor was created by the researchers using Vizard, with a ground or virtual height condition (3-m, 6-m, or 9-m height, as seen in Figure 1).

To incorporate the VR sensory manipulation and mechanical perturbation and to minimize the order of heights affecting the experiment results, the height changes were provided in a pseudo-randomized pattern to the participants and the toe-down perturbation was induced at each height during the second and third blocks. Thus, each participant experienced all height conditions with and without mechanical perturbation. Based on the height and perturbation onset, all conditions within the four blocks were further classified into four overarching conditions, which were ground condition, height condition, ground with perturbation condition, and height with perturbation condition. These four overarching conditions were used to label and perform grand averages for primary outcome measurements and used as condition levels in subsequent statistical analyses.

### 2.4 Data acquisition and pre-processing

High-density EEG data from a 64-channel active system (actiChamp system, Brain Vision LLC, Morrisville, NC USA) were recorded during each condition to assess the cortical activations of the participants. The EEG system consisted of a cap plus two electrooculography (EOG) electrodes, temporal to and inferior to the right eye, two EMG electrodes on the right and left trapezius, one reference electrode on the right mastoid, and a ground electrode on the left mastoid. Data were recorded at 1,000 Hz and referenced to the average of the left and right mastoid electrodes. The positions of the EEG sensors on the head were based and modified from international 10–10 systems. For EMG, bilateral ankle flexor and extensor muscle activities were recorded with Trigno wireless EMG system (Delsys Inc, Natick MA, USA) with a sampling rate of 1,926 Hz. EMG sensors were applied to bilateral tibialis anterior (TA), gastrocnemius medial head (GAM), gastrocnemius lateral head (GAL), and soleus (S) muscles. The SECRS research module was used to acquire COP data with a 100 Hz sampling rate while providing toe-down perturbations synchronized with VR environment.

All data processing was performed with customized MATLAB scripts (MathWorks, Natick, MA, USA). Raw EEG data were high-pass filtered at 1Hz to remove drift and low-pass filtered at 55 Hz to remove line noise. A band-specific filter for 60 Hz was applied due to the environment line noise in the lab. EEG artifacts associated with eye and other muscle movements were removed using independent component analysis (ICA). Based on the topography, spectra, and trial-to-trial characteristics of ICA components, good fit ICA components were selected and used to generate back-projected EEG data, referred to as clean EEG. The clean EEG data were further trimmed based on the synchronization time stamps into segmented EEG data corresponding to each VR-HCT condition in each block for each participant. EMG raw data were first resampled to 1,000 Hz (the EEG sampling rate) and then low pass filtered with a cutoff frequency of 400 Hz (Mello et al., 2007). After being corrected by the average signal over the entire recording, the EMG data were rectified. The signals from GAL and GAM were grand averaged as one gastrocnemius signal (GA). The EMG data were further segmented based on the timestamps of the onset and offset of each VR-HCT condition in each block for each participant. COP raw data were low pass filtered using a Butterworth filter with a cutoff frequency of 10 Hz. Then, the COP data were segmented based on the 30-s conditions in each block. The average anterior-posterior and medial-lateral positions in each segmented block in each block were calculated as the standing center point and used to correct the COP raw data in the corresponding block. The corrected COP data were referred to as segmented COP data.

### 2.5 Primary measures

#### 2.5.1 Corticomuscular coherence assessment

For each VR-HCT condition (30 s), the power spectral density of EEG and rectified EMG was computed using Welch's method with 500 ms non-overlapping Hanning windows (Gwin and Ferris, 2012). CMC was evaluated using the magnitude square coherence for each EEG channel/EMG channel paired using Equation 1:


(1) $$\begin{array}{l} coh_{c1c2}\left(f\right)=\frac{|s_{c1c2}\left(f\right){|}^{2}}{s_{c1c1}\left(f\right)\cdot s_{c2c2}\left(f\right)} \end{array}$$

where *s*_*c*1*c*1_ and *s*_*c*2*c*2_ are the auto-spectra of each signal; *s*_*c*1*c*2_ is the cross-spectra; and (f) denote the band of interest, which were β (13–30 Hz) and γ (31–45 Hz). CMC was calculated for each paired EEG and EMG signal and was further grand averaged based on corresponding cortical regions with specific ankle muscles as follows: frontal (Fz, F5, and F6), central (Cz, C5, and C6), parietal (Pz, P5, and P6), and occipital (Oz, O5, and O6).

#### 2.5.2 Postural control assessment

Based on the segmented COP data, the following COP indices were extracted to describe the postural control abilities. The range of COP displacement was defined as the differences between the maximal COP positions, which were extracted in the anteroposterior (*R*_*AP*_) and mediolateral (*R*_*ML*_) directions separately. The total root mean square (RMS) of COP displacements was calculated using


(2) $$\begin{array}{l} RMS=\sqrt{\frac{1}{n}\overset{n}{\underset{i=1}{\sum }}x_{i}^{2}} \end{array}$$

where *n* denotes the number of measurements, and *x*_*i*_ denotes each displacement value. The RMS of COP displacement from each position to the center point (*COP*_*rms*_), COP displacement in the anterior-posterior direction (*COP*_*rmsAP*_), and COP displacement in medial-lateral direction (*COP*_*rmsML*_) was extracted. Sway energy was also calculated in each unique condition in each block. Specifically, the following equation was used:


(3) $$\begin{array}{l} SwayEnergy=C1\times RMS\left(PY^{\prime }\right)+C2\times RMS\left(PY^{\prime \prime }\right) \end{array}$$

where the constants *C*1 and *C*2 are defined as *C*1 = 1/(*inches*/*second*) and *C*2 = 0.025/(*inches*/*second*^2^). While *PY* denotes COP displacement in AP direction, *PY*′ denotes the first derivative of *PY*, and *PY*^′′^ denotes the second derivative of *PY*. This equation was based on the SECRS system equations that are used to estimate the unitless sway energy in the clinical adaption test (Mcguirk, 2005).

### 2.6 Secondary measures

Furthermore, baseline physical, cognitive, and psychological function was evaluated to help control for potential covariates in cortical activation and postural control. Before the VR-HCT test, the MiniBESTest battery was conducted to evaluate the functional balance of the participants. The MiniBESTest test score was used to quantify functional balance. Participants' executive function was evaluated using the Trail Making Test (TMT). The fall risk of the participants was assessed by the Falls Efficacy Scale-International (FES-I). The fear of height was assessed by the Acrophobia Questionnaire-Anxiety Subscale. This questionnaire is a 20-item self-report questionnaire that asks participants to rate their anxiety related to height-relevant situations using a 0 (not at all anxious; calm and relaxed) to 6 (extremely anxious) scale. A Tai Chi experience questionnaire was given to participants during the pre-screening phone interview. The questions include (1) “Have you practiced Tai Chi or Taijiquan before?”; (2) “Are you currently actively practicing Tai Chi?”; (3) “How long have you practiced Tai Chi? in weeks or years.”; (4) “In total, how many hours have you practiced Tai Chi?”; (5) “Currently, on average, how many hours do you practice Tai Chi every week?”. Questions three to five were used to quantify the Tai Chi practitioner's experience into total hours.

### 2.7 Statistical analysis

All the statistical analyses were performed using R (R 4.4.0, RStudio 2024.12.0+467). There were three sets of statistical analyses performed to answer the research questions. The one-way ANOVA was used to test the cohort demographic differences and secondary measurement differences. For primary outcome measurements, outliers were detected and removed using an Interquartile Range (IQR) method with a 1.5 IQR cutoff. To achieve residual normality, log or square-root data transformation were performed based on the direction and level of skewness of the data set. A positive square-root transformation was used on sway energy, anteroposterior COP RMS, and β and γ CMC. Linear mixed effect models (LMMs) were used to identify the cohort differences for cortical activities and postural control performance, using subject as a random factor, and cohort (young adult, older adult, and Tai Chi practice groups), condition (ground, height, ground with perturbation, and height with perturbation), cortical region (frontal, central, parietal, and occipital), and muscle (TA, GAM, GAL, and S) as fixed effects. Model significance was evaluated using the likelihood ratio test. Linear mixed model assumptions were confirmed using visual inspection. The best model identified included all one-way factors, two-way interactions with cohort, and cohort × muscle × condition three-way interaction. Least square means *post-hoc* comparisons were performed using a *p*-value of <0.05 and reported in Supplementary material. Moreover, Spearman's correlations were used to investigate the relationship between CMC and COP indices in each test.

## 3 Results

**Figure 2:**
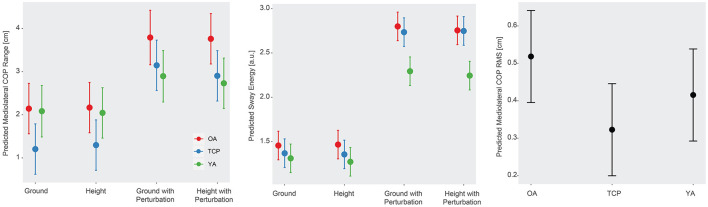
**(Left)** Predicted mediolateral COP range across conditions and cohorts, **(Center)** sway energy across conditions and cohorts, and **(Right)** mediolateral COP RMS changes across cohorts.

Statistically significant differences in anteroposterior COP range (*R*_*AP*_), mediolateral COP range (*R*_*ML*_), anteroposterior COP RMS (*COP*_*rmsAP*_), mediolateral COP RMS (*COP*_*rmsML*_), sway energy, and overall COP RMS (*COP*_*rms*_) were observed across different conditions (*p* < 0.01, Figure 2). In particular, higher *R*_*AP*_, *R*_*ML*_, *COP*_*rmsAP*_, *COP*_*rmsML*_, sway energy, and *COP*_*rms*_ were found in ground and height with perturbation conditions, relative to ground condition. In addition, a significant cohort effect on *COP*_*rmsML*_ was observed (*p* < 0.05), with Tai Chi practitioners demonstrating a lower *COP*_*rmsML*_ than older adults. Furthermore, a statistically significant cohort by condition interaction effect was observed in *R*_*ML*_ (*p* < 0.05) and sway energy (*p* < 0.01), such that older adults had a higher *R*_*ML*_ and sway energy than young adults in the height with perturbation condition, relative to ground condition (Figure 2). No other statistically significant effects were detected in postural control metrics.

### 3.1 Corticomuscular coherence

The β range coherence peaks occurred at 21.5 ± 5.39 Hz for young adults, 21.6 ± 5.37 Hz for older adults, and 21.7 ± 5.38 Hz for Tai Chi practitioners. The γ range coherence peaks occurred at 37.9 ± 4.48 Hz for young adults, 38.0 ± 4.50 Hz for older adults, and 28.0 ± 4.49 Hz for Tai Chi practitioners. These frequency peaks were not harmonics of each other across the frequency ranges. Peaks in the β EMG spectral power occurred at 18.0 ± 5.29 Hz for young adults, 16.0 ± 4.63 Hz for older adults, and 16.4 ± 5.26 Hz for Tai Chi practitioners. Peaks in the γ EMG spectral power occurred at 35.6 ± 4.27 Hz for young adults, 35.3 ± 4.29 Hz for older adults, and 34.7 ± 3.87 Hz for Tai Chi practitioners. Peaks in the β EEG spectral power occurred at 14.3 ± 3.22 Hz for young adults, 15.5 ± 2.88 Hz for older adults, and 15.2 ± 2.94 Hz for Tai Chi practitioners. Peaks in the γ EEG spectral power occurred at 31.9 ± 1.72 Hz for young adults, 32.0 ± 1.66 Hz for older adults, and 31.7 ± 1.46 Hz for Tai Chi practitioners (Supplementary Table S1).

#### 3.1.1 β corticomuscular coherence

**Figure 3:**
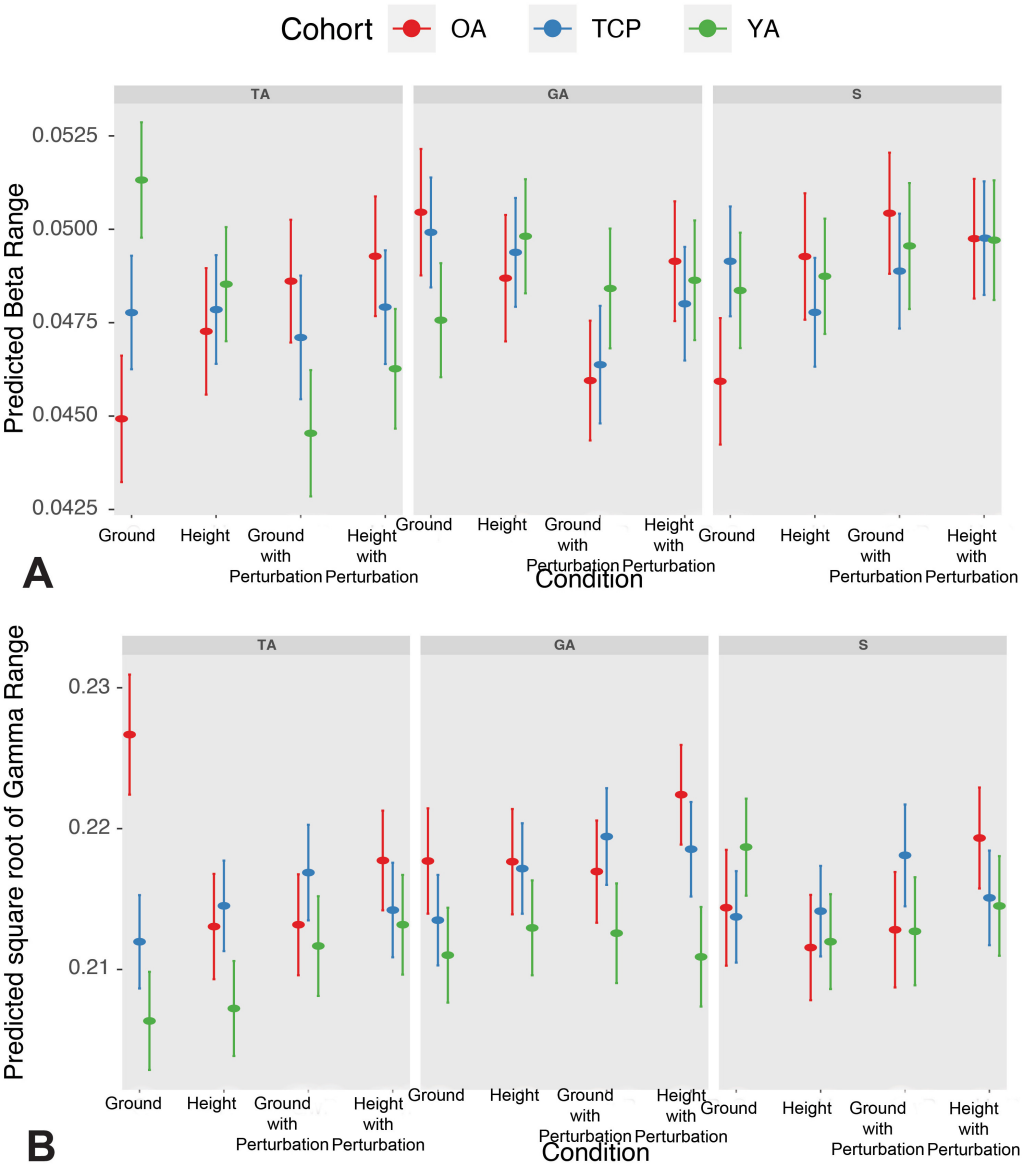
**(A)** Predicted β CMC range across conditions and cohorts; and **(B)** Predicted square root of γ CMC range across conditions and cohorts. YA, young adult; OA, older adult; TCP, older adult with Tai Chi practice; TA, tibialis anterior; GA, gastrocnemius; S, soleus.

Overall, a statistically significant condition effect (*p* < 0.05), muscle effect (*p* < 0.001), cohort-condition interaction effect (*p* < 0.01), condition-muscle interaction effect (*p* < 0.001), and cohort-muscle-condition interaction effect (*p* < 0.001) were observed on β CMCbased on linear mixed models (Figure 3, Table 2).

**Table 2:** Linear mixed effect model type III analysis of variance table with Satterthwaite's method.

**Factor**	***F* value**	***p*-value**	**Signif**.
β **Corticomuscular coherence:**			
Cohort	0.0434	0.9576	
Condition	2.8269	0.0375	^*^
Muscle	9.9350	0.0001	^***^
Regions	0.3117	0.8169	
Cohort: Condition	3.2049	0.0040	^**^
Cohort: Regions	0.2044	0.9755	
Cohort: Muscle	0.0515	0.9950	
Condition: Muscle	5.1341	0.0000	^***^
Cohort: Condition: Muscle	5.8546	0.0000	^***^
γ **Corticomuscular coherence:**			
Cohort	9.5949	0.0007	^***^
Condition	4.4722	0.0039	^**^
Muscle	4.4705	0.0116	^*^
Regions	0.0608	0.9804	
Cohort: condition	5.7160	0.0000	^***^
Cohort: regions	0.4541	0.8424	
Cohort: muscle	6.3317	0.0000	^***^
Condition: muscle	1.6507	0.1298	
Cohort: condition: muscle	3.1125	0.0002	^***^

*Significance codes:* ^***^p < 0.001, ^**^p < 0.01, ^*^p < 0.05.

##### 3.1.1.1 *Post-hoc* results

Based on *post-hoc* contrasts, increased β CMC was observed in height with perturbation relative to ground with perturbation conditions and in gastrocnemius and soleus muscles relative to tibialis anterior (see Supplementary Table S2). In particular, older adults demonstrated increased β CMC in height with perturbation relative to ground conditions. Furthermore, during ground conditions, decreased β CMC was observed in older adults, relative to both young adults and Tai Chi practitioners. Further examination of cohort differences by muscle and condition demonstrated that tibialis anterior β CMC during ground conditions significantly increased in Tai Chi practitioners and young adults, relative to older adults, and in young adults relative to Tai Chi practitioners. In contrast, tibialis anterior β CMC during ground and height with perturbation conditions significantly decreased in young adults relative to older adults and in young adults relative to Tai Chi practitioners in ground with perturbation conditions. Furthermore, in gastrocnemius β CMC during ground and ground with perturbation conditions significantly decreased in young adults relative to older adults and in young adults relative to Tai Chi practitioners in ground conditions. Finally, in soleus β CMC during ground condition significantly increased in Tai Chi practitioners and young adults relative to older adults.

#### 3.1.2 γ corticomuscular coherence

Overall, a statistically significant cohort effect (*p* < 0.001), condition effect (*p* < 0.01), muscle effect (*p* < 0.05), cohort-condition interaction effect (*p* < 0.001), cohort-muscle interaction effect (*p* < 0.001), and cohort-muscle-condition interaction effect (*p* < 0.001) was observed on square-root transformed γ CMC.

##### 3.1.2.1 *Post-hoc* results

Based on *post-hoc* contrasts, increased γ CMC was found in both older adults and Tai Chi practitioners, relative to young adults (see Supplementary Table S3). Furthermore, increased γ CMC was observed in height with perturbation relative to height conditions and in gastrocnemius relative to tibialis anterior. In particular, older adults demonstrated increased γ CMC in height with perturbation relative to height and ground with perturbation conditions, while decreased γ CMC was observed in height and ground with perturbation conditions, relative to ground conditions. Furthermore, Tai chi practitioners demonstrated increased γ CMC in ground with perturbation conditions relative to ground conditions. Furthermore, during both ground and height with perturbation conditions, increased γ CMC was observed in older adults, relative to both young adults and Tai Chi practitioners. In addition, in both height and ground with perturbation conditions, Tai Chi practitioners had increased γ CMC, relative to young adults. Further examination of cohort differences by muscle and condition demonstrated that tibialis anterior γ CMC during ground conditions significantly decreased in Tai Chi practitioners and young adults, relative to older adults, and in young adults relative to Tai Chi practitioners. Similarly, tibialis anterior γ CMC during height conditions significantly decreased in younger adults, relative to older adults and Tai Chi practitioners, and in younger adults, relative to Tai Chi practitioners in ground with perturbation conditions.

### 3.2 Correlation between corticomuscular coherence and postural control

The correlation between corticomuscular coherence meausures and postural control measures that was found to have significant cohort effects or interactions was examined using Spearman's correlation. A negative association was found between β CMC and *COP*_*rmsML*_ in the ground condition (ρ = -0.120, *p* < 0.05). Specifically, subgroup analysis found a negative correlation in Tai Chi practitioners (ρ = –0.31, *p* < 0.01). A negative correlation was found between γ CMC and *COP*_*rmsML*_ in the ground condition (ρ = –0.156, *p* < 0.01). Specifically, subgroup analysis found a negative correlation in older adults (ρ = –0.38, *p* < 0.01) and Tai Chi practitioners (ρ=-0.39, *p* < 0.01) and a positive correlation in young adults (ρ = 0.3, *p* < 0.01). A negative correlation was found between γ CMC and *R*_*ML*_ in the ground condition (ρ = –0.115, *p* < 0.05). Specifically, subgroup analysis found a negative correlation in older adults (ρ = –0.28, *p* < 0.01) and Tai Chi practitioners (ρ = –0.39, *p* < 0.01) and a positive correlation in young adults (ρ=0.28, *p* < 0.01). A positive correlation was found between γ CMC and sway energy in ground with perturbation condition (ρ = 0.275, *p* < 0.001) and in height with perturbation condition (ρ = 0.251, *p* < 0.001). Specifically, subgroup analysis found a positive correlation in older adults in the ground with perturbation condition (ρ = 0.41, *p* < 0.01) and in older adults in height with perturbation condition (ρ = 0.24, *p* < 0.05).

## 4 Discussion

This study furthered our understanding of the effects of Tai Chi practice and healthy aging on cortical and neuromuscular function, as evaluated by CMC, while standing in balance-demanding virtual reality environments. Partly consistent with our hypotheses, we found that (1) compared to young adults and Tai Chi practitioners, older adults demonstrated significantly lower β CMC in ground conditions and higher γ CMC in both ground and height with perturbation conditions; (2) β CMC was significantly correlated with center of pressure indices in ground conditions; and (3) γ CMC was significantly correlated with center of pressure indices in ground, ground with perturbation, and height with perturbation conditions, consistent with observed changes in β and γ range CMC in lower extremity isometric and isotonic exercises (Gwin and Ferris, 2012).

We found older adults, relative to young adults and Tai Chi practitioners, to have decreased β CMC in ground conditions, which was consistent with decreases in β CMC previously seen in older adults (Bayram et al., 2015; Kamp et al., 2013). Furthermore, relative to young adults and Tai Chi practitioners, older adults were found to have increased γ CMC in both ground and height with perturbation conditions, consistent with tasks requiring increased sensorimotor integration (Omlor et al., 2007). In addition, Tai Chi practitioners had increased γ CMC, relative to young adults, in both height and ground with perturbation conditions, which may reflect an adaptive change in Tai Chi practitioners. Combined, these findings suggest aging might be associated with loss of isometric ankle strategy in upright postural control, and this effect might be partly overcome by practicing Tai Chi as an older adult. As β CMC was associated with isometric contraction and constant force output, and γ CMC was related to strong contraction and isokinetic contraction (Gwin and Ferris, 2012), these relevant CMC changes indicated stronger contraction and less isometric contraction presented in older adults compared to Tai Chi practitioners and young adults. In this study, as all participants were instructed to stand as still as possible, an isometric ankle strategy with minimal lower limb joint flexion and extension was expected to maintain upright postural. However, the significantly higher γ CMC in soleus and higher γ and β CMC in gastrocnemius relative to tibialis anterior indicated a potential shift to voluntary plantarflexor control to maintain balance during quiet standing in older adults. Alternatively, Tai Chi practice may contribute to more efficient cortical control by enhancing temporal precision, focused engagement, and the economical structuring of movement, a theoretical possibility supported by prior accounts of resource-sensitive motor regulation in aging and under energetic constraints (Schumann et al., 2022; Tomporowski, 2008). This effect may arise from two interrelated processes: the targeted mobilization of cognitive resources for motor coordination, and the refinement of control strategies that optimize the alternation between muscular tension and relaxation, thereby supporting a dynamic and context-sensitive regulation of effort within the movement flow (Schumann et al., 2022).

Confirming the second hypothesis, in ground condition, higher β CMC was associated with lower postural sway (*COP*_*rmsML*_), and subgroup analyses suggest this relationship was enhanced and significant in TCP while there was non-relationship in older adults and young adults. At the same time, higher γ CMC was associated with lower postural sway and COP displacement range (*R*_*ML*_) in the ground condition. However, while higher γ CMC was associated with lower postural sway and COP displacement range in both older adults and Tai Chi practitioners, higher γ CMC was associated with higher postural sway and COP displacement range in young adults. These results suggest that corticomuscular coupling might be essential in postural stability, especially during quiet standing in older adults, which might be explained by compensation theories of aging (Seidler et al., 2010). Specifically, the opposite directionalities in the γ CMC and postural stability relationships between young adults and older adults' groups may suggest γ corticomuscular coupling was engaged in regulating the postural stability in older adults. γ corticomuscular coupling might be related to the initiation of voluntary movement in ML direction in young adults. This is supported by the similar postural sway level that was found in young adults compared to Tai Chi practitioners, and larger COP displacement ranges that were found in YA compared to Tai Chi practitioners. Moreover, while older adults and Tai Chi practitioners' CMC γ increased along with reduced postural sway, a significantly higher postural sway was still found in older adults compared to Tai Chi practitioners. In addition, as the correlation between β CMC and postural sway was only significant in Tai Chi practitioners, combined with the significant differences between older adults, Tai Chi practitioners, and young adults in β CMC levels discussed above, the ability to maintain β corticomuscular coupling might be a key for successfully maintaining balance while standing in older adults.

Confirming the third hypothesis, γ CMC was positively correlated with COP sway energy in both ground with perturbation condition and height with perturbation condition. Interestingly, subgroup analyses suggest these relationships were enhanced and only significant in older adults while there was non-relationship in Tai Chi practitioners and young adults. These results suggest a potential unsuccessful γ cortical coupling compensation might be presented in older adults to regulate postural stability under perturbation. As sway energy was calculated based on RMS of the first and second derivative of COP position along AP direction, sway energy describes the ability to regulate the movement velocity in AP direction. Higher sway energy presented during perturbation conditions indicated that perturbations induced higher COP velocity and acceleration changes in AP direction in older adults and Tai Chi practitioners than young adults.

The positive relationship between γ CMC and sway energy might indicate older adults failed to control the COP velocity and accelerations. As previous study suggested that γ CMC is associated with isokinetic contraction and strong muscle contractions (Gwin and Ferris, 2012), our results suggested older adults might had strong muscle contractions which involved lower limb joints' angle changes, which might be linked with passively response to the irregulated COP movement in AP direction under perturbation. On the other hand, as these relationships were not established in young adults and Tai Chi practitioners, they were not engaging in γ corticomuscular coupling to regulate COP movement under perturbation conditions. The positive relationship between γ corticomuscular coupling and COP sway energy in older adults also suggests that the high γ CMC was unsuccessful compensations for control of the COP movement, which can be explained by the aging-related unsuccessful compensation behavior under the framework of the Hemispheric Asymmetry Reduction in Older Adults (HAROLD) model (Cabeza and Dennis, 2013).

There are several limitations of this study. First, the anteroposterior COP features might be dominated by the pre-programed perturbation profile, thus failed to detect the impacts other than the condition effect in the LMMs. Specifically, during the perturbation conditions, the period of perturbation onset (~3 s) and the perturbation offset period (~27 s) were not separated, which might allow the preprogrammed mechanical perturbation amplitude to overwrite the underlying cohort differences. The drastic effect during the 3 s could overwrite the underlying cohort differences, specifically when the indices were closely linked with the absolute position, such as range of position and total COP sway. Future analysis should segment the perturbation condition further to perturbation onset or offset period. It might help to provide additional insight into cohort differences in response to perturbation in the anterior-posterior direction. Second, as a cohort study, the Tai Chi practitioners has a large range of Tai Chi practice time and a large variance of Tai Chi style of practice, which might introduce potential confounding variables into the data analysis. In addition, the increased time needed to complete the trail making test—form A suggests differences in complex attention or selective decline in cognitive and attentional functions among the Tai Chi practitioners (McMorris, 2016) but may also reflect age-related changes in motoric performance, given the strong contribution of age on TMT A and B performance (Tombaugh, 2004). Future work should implement a randomized clinical trial design to minimize these confounding factors and utilize repeated observations to improve design-level constraints and allow for the evaluation of differential effectiveness in Tai Chi practice.

## 5 Conclusion

This study investigated the corticomuscular coherence between cortical areas and ankle plantarflexor and dorsiflexor muscles during novel virtual reality height control tests. To our knowledge, this is the first study to investigate the effect of Tai Chi and older age on corticomuscular coherence during standing postural challenges. In particular, this study demonstrated how corticomuscular coherence patterns may reflect compensatory mechanisms in aging and how these might be modifiable through structured movement practice. Thus, through the integration of modern technologies, such as virtual reality and neuromechanics, with traditional movement practices, we can refine our conceptual understanding of embodied cognition and adaptive motor control. While the dose-response relationship of Tai Chi training and temporal dynamics of cortical recruitment after Tai Chi practice remain open questions, these findings support Tai Chi as a low-impact, accessible, and culturally meaningful, form of physical activity that may help stabilize postural control in older adults.

## Data Availability

The raw data supporting the conclusions of this article will be made available by the authors, without undue reservation.
